# Changes in urinary stable nitrogen isotope ratios during controlled short-term energy deficit: a proof-of-principle analysis

**DOI:** 10.1007/s00394-023-03320-8

**Published:** 2024-01-19

**Authors:** Paulina Wasserfurth, Frank Huelsemann, Karsten Koehler

**Affiliations:** 1 Department of Health and Sport Sciences, TUM School of Medicine and Health, Technical University of Munich, Munich, Germany; 2 Institute of Biochemistry, German Sport University, Cologne, Germany

**Keywords:** Low energy availability, Weight loss, Biomarkers

## Abstract

**Purpose:**

Stable isotope ratios of nitrogen (δ^15^N) have previously been shown to increase in human hair during periods of catabolism. The goal of this study was to assess changes in δ^15^N in urinary urea (δ^15^N_urea_) and Δ^15^N during a short-term controlled energy deficit.

**Methods:**

We analyzed samples from 6 recreationally active men (25 ± 1 years, BMI: 23.5 ± 0.6 kg/m^2^) who participated in a repeated measures cross-over study involving 4 days of energy deficit (ED, ~ 15 kcal/kg FFM) without and with exercise (ED-EX, ED + EX) and control conditions in energy balance (CON-EX, CON + EX). δ^15^N_urea_ was analyzed from urine samples, and Δ^15^N was calculated as δ^15^N_urea_–δ^15^N_diet_, with δ^15^N_diet_ obtained from diet prescriptions.

**Results:**

δ^15^N_urea_ was significantly elevated in ED-EX (4.4 ± 0.2‰) when compared to CON-EX (3.7 ± 0.1‰; *p* = 0.026) and CON + EX (3.34 ± 0.13‰, *p* = 0.001). As a consequence, Δ^15^N was positive in ED-EX (0.2 ± 0.2‰) and remained negative in ED + EX (− 0.6 ± 0.5‰), CON-EX (− 1.0 ± 0.2) and CON + EX (− 1.1 ± 0.2). Differences in Δ^15^N were significant between ED-EX and CON-EX (*p* = 0.005) and ED-EX and CON + EX (*p* = 0.006).

**Conclusion:**

Our results suggest that δ^15^N_urea_ and subsequently Δ^15^N are responsive to a short-term energy deficit, likely due to increased amino acid oxidation to meet energy demands and preferable elimination of ^14^N.

## Introduction

Body protein represents the largest reservoir of amino acids (AAs) and nitrogen in humans. On a daily basis, there is a constant uptake and release of AAs from different tissues to the free AA pool, which ensures constant supply of AAs for protein synthesis and availability of carbon and nitrogen for other metabolic processes, such as gluconeogenesis or synthesis of neurotransmitters [1]. These metabolic fluxes are regulated by dietary factors, such as protein and energy intake and change with altering physiological demands. For example, in an energy deficit (ED), when energy requirements are met by the breakdown of body energy stores, increased proteolysis ensures supply with AAs for gluconeogenesis [2]. Sustained energy deficits are associated with a negative nitrogen balance and the loss of body protein, as approximately 25% of weight loss is accounted by fat-free mass (FFM) [3].

One biomarker, which appears to reflect periods of negative nitrogen balance, is the ratio of naturally occurring stable nitrogen isotopes (δ^15^N) [4, 5]. Generally, δ^15^N in body tissues reflects the isotope ratio of the diet, allowing for the classification of the trophic level in animals or the differentiation between plant- vs. animal-based diet in humans [6, 7]. δ^15^N has also been widely used to characterize dietary habits in archeological, ecological and epidemiological studies [8]. However, when compared to the diet, body tissues are typically enriched in the heavier isotope ^15^N, as the lighter isotope ^14^N is preferably metabolized and excreted due to discrimination against ^15^N during trans- and deamination [9].

At the same time, δ^15^N and Δ^15^N are impacted by physiological demands. Evidence shows that δ^15^N increases in various tissues in both animals and humans in response to inadequate dietary intake or starvation [10–13]. In humans, most studies have measured δ^15^N in hair (δ^15^N_hair_), which has been shown to be consistently elevated during periods of extreme energy deficiency, such as starvation or anorexia nervosa [13–15]. We have previously measured δ^15^N_hair_ over the course of a 25-day expedition, during which food intake was strongly limited and substantial weight loss occurred [16]. Anecdotally, δ^15^N_hair_ was highest when the energy deficit was greatest [16, 17]. Similarly, δ^15^N_hair_ also increased in pregnant women experiencing weight loss due to morning sickness [18], and data from animal studies also show that δ^15^N increases in various tissues in response to prolonged energy deficiency [10–12, 19]. Although the exact mechanisms for the elevation in δ^15^N during energy deficiency remain to be elucidated, it has been proposed that the increased breakdown of body protein leads to release of ^15^N-enriched AAs, which are subsequently recycled [4]. However, using data from multiple tissues and nitrogen pools obtained from rats, Poupin et al. demonstrated that δ^15^N of tissues is modulated by several metabolic pathways [20].

Despite successful characterization of periods of catabolism in humans, δ^15^N_hair_ only allows for retrospective analysis with a resolution of days to weeks [21, 22]. Given that urea is the end-product of AA catabolism and reflective of AA oxidation, measurements of δ^15^N in urinary urea (δ^15^N_urea_) might serve as a more immediate alternative which could provide greater and timely resolution. In fact, within the first few days of fasting, excretion of urinary urea increases as a consequence of increased protein degradation and subsequent use of AA as substrates for gluconeogenesis [23].

Preliminary data from our group suggest that δ^15^N_urea_ is highly sensitive to short-term changes in dietary intakes and does not require large sample sizes [21]. Yet, it has to be confirmed that δ^15^N_urea_ is also impacted during short-term ED. To assess the potential of δ^15^N_urea_ and Δ^15^N as indicators of catabolism, we performed a proof-of principle analysis on samples from a previously published study employing an ED with and without exercise [24]. Our primary working hypotheses was that δ^15^N_urea_ and consequently Δ^15^N would increase during ED, indicating increased rates of AA oxidation in the energy-deficient state.

## Materials and methods

### Experimental design

**Fig. 1:**
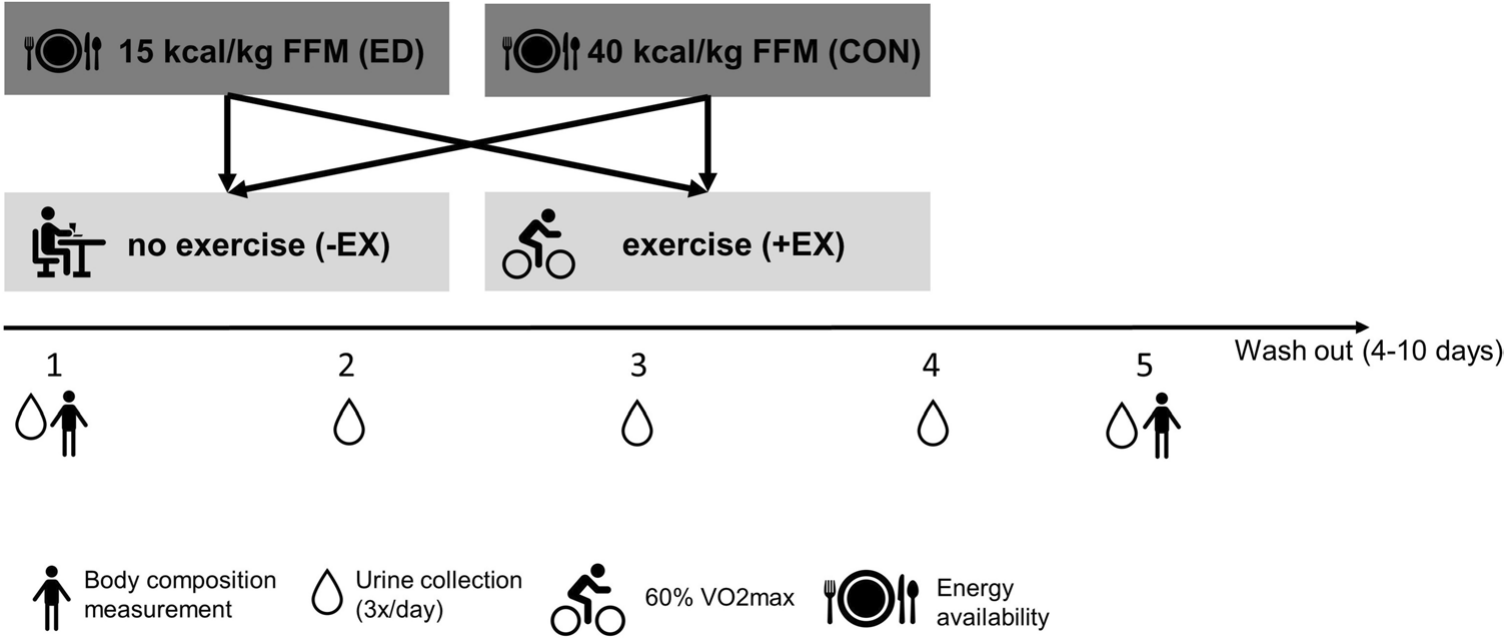
Overview of the study design. In this four-way cross-over study, participants underwent 4 days of either energy deficit (ED) or energy balance (CON) without (-EX) or with exercise (+ EX). Energy deficit and energy balance were operationally defined as an energy availability of 15 kcal/kg/fat-free mass (FFM) or 40 kcal/kg/FFM, respectively

For the present investigation, we analyzed samples from a randomized controlled trial assessing the impact of an ED with and without exercise on metabolic and behavioral adaptations [24, 25]. To test the independent and combined effects of an ED and exercise, the study was conducted using a four-way cross-over design during which participants underwent two 4-day conditions of ED and two 4-day control conditions in energy balance (CON) (Fig. 1). During one ED and one CON condition, participants conducted aerobic exercise (ED + EX; CON + EX). During the other ED and CON conditions, no exercise was conducted (ED-EX; CON-EX). To generate isocaloric conditions within ED and CON, dietary energy intake was adjusted for the energy expended during exercise. The order of the experimental conditions was randomly assigned. After each condition, participants underwent wash out periods of ad libitum food intake. Wash-out periods were set to at least 4 days following CON and at least 10 days following ED conditions. The study was approved by the ethical review board of the German Sport University Cologne (no approval number was given) and was conducted in accordance with the Declaration of Helsinki.

### Participants

Participants were recruited from the university community in accordance with the following inclusion criteria: male, 18–30 years of age, ≥ 3 h/week aerobic exercise, normal (body-mass index: 19–25 kg/m^2^; body fat percentage ≤15%), and stable body weight (± 3 kg during the past 6 months). Exclusion criteria were: smoking, infectious disease within the past 4 weeks, intake of medication that could influence the study outcomes, cardiovascular disease or orthopedic impairment interfering with conducting exercise, diabetes or history of a clinical eating disorder. All participants provided written informed consent prior to participation in the study.

### Body weight and body composition

At baseline and the beginning and end of each condition, body weight and body composition were assessed with a bioimpedance scale (Tanita BC 418 MA, Tanita, Amsterdam, The Netherlands). All measurements were carried out in the morning with participants in a fasted (≥ 10 h) and well-hydrated state [24].

### Diet prescription

Energy deficit was operationally achieved by reducing energy availability, defined as dietary energy intake minus exercise energy expenditure, to 15 kcal/kg FFM, which represents approximately 50% of basal energy requirements [26]. Energy balance was assumed at an energy availability of 40 kcal/kg FFM [27]. Detailed meal plans were developed for each participant, based on individual dietary habits and food preferences. The meal plans served to ensure energy intake was in accordance with the respective conditions. Regarding macronutrient distribution, it was aimed to keep the intakes within the range of the recommendations of the German Nutrition Association (50–55% carbohydrates, 30–35% fat, 10–15% protein) while also assuring that protein intake at least met the recommended daily allowance for adults (0.8 g/kg) [28]. During all conditions, participants weighed all foods consumed as well as leftovers with a food scale and reported their intake daily. Analysis of food consumed occurred on a daily basis (EBIS pro version 7.0, University of Hohenheim, Stuttgart, Germany, 2005) and meal plans were adjusted if reported and prescribed intake differed by ≥ 50 kcal.

### Exercise prescription

During exercise conditions, participants performed supervised exercise on the bicycle ergometer at 60% of their VO_2peak_ until an exercise energy expenditure of 15 kcal/kg FFM was achieved. Outside of the intervention, participants were instructed to abstain from any additional exercise, which was monitored with an activity tracker (SenseWear Pro3 armband, Bodymedia, Pittsburgh, USA).

### Stable nitrogen isotope ratio analysis and calculations

For analysis of δ^15^N_urea_, participants were asked to collect three urine samples per day. Samples were collected in the morning in a fasted state as well as in the early afternoon and at bedtime. Participants were instructed to record the time of sample collection and the total urine volume. A 50-mL aliquot of each sample was stored in the participants’ refrigerators until delivery to the laboratory, which occurred within 24–48 h. For precipitation of urinary urea, 250 μL of urine were acidified with 375 μL of glacial acetic acid (analytical grade; Merck, Darmstadt, Germany) and 375 μL 10% (w/w) xanthydrol (Sigma-Aldrich, Steinheim, Germany) in methanol (analytical grade; VWR, Darmstadt, Germany) were added and stored at 6 °C for 12 h [21]. Thereafter, the mixture was centrifuged, the supernatant discarded and the precipitate (*N,N′*-dixanthylurea) washed twice with methanol/water (2:1 v/v). *N,N′*-dixanthylurea was dried overnight in vacuum over phosphorus pentoxide. For analysis, ~ 500 μg *N,N′*-dixanthylurea were weighed into tin capsules and δ^15^N of urea was assessed using elemental analysis–isotope ratio mass spectrometry. Isotope ratios are expressed relative to atmospheric nitrogen (AIR). The elemental analyzer (Eurovektor EA 3000, Hekatech, Wegberg, Germany) was coupled to a Delta C continuous-flow isotope ratio mass spectrometer (Thermo Fisher Scientific, Bremen, Germany). The working standard gas (N2, purity 5.0, Linde, Munich, Germany) and the working standard (creatine-monohydrate, AlzChem, Trostberg, Germany) were scale calibrated using IAEA-N-1 (+ 0.4 ‰) and IAEA-N-2 (+ 20.3 ‰) for δ^15^N values (IAEA, Vienna, Austria). All measurements were carried out in triplicates and the standard deviation for triplicate measure of the working standard was ± 0.2‰. Instrument stability and analytical performance were checked by regular analysis of the working standard and zero blanks. For the present analysis, data from the three daily samples were aggregated into a daily average of δ^15^N_urea_, and δ^15^N_urea_ was further averaged across each of the 4-day study condition. Δ^15^N was calculated as the difference between δ^15^N_urea_ and dietary nitrogen isotope ratio (δ^15^N_diet_), which was obtained from the prescribed diet as previously described [29].

### Statistical analysis

Statistical analyses were performed with R (version 4.1.1). If not stated otherwise, data are reported as mean ± standard error of the mean (SEM). To account for the small sample size and missing data from one participant, we used linear models to identify changes within conditions, between conditions, and over time. The subject identifier was included in all models to account for the repeated measures. If a trend was observed (*p* < 0.1), paired one sided t-tests were performed as post-hoc analyses and p-values were adjusted for multiple testing using Holm-correction. Statistical significance was set at < 0.05. Effect sizes were calculated from the difference of means and standard deviation of those differences, with *d* = 0.2 considered as small, *d* = 0.5 as medium and *d* > 0.8 as large effect [30]. To evaluate the impact of potential confounders on Δ^15^N, multiple linear regression analysis was performed using Δ^15^N as dependent and protein intake (in g/day), energy availability (in kcal/kg FFM) and the interaction as independent variables. Subject identifiers were included in all models and adjusted R^2^ was interpreted. The standardized coefficient (β) was obtained from multiple linear regression with Z-transformed variables. Sample size was predetermined through power calculations performed for the primary analysis [24].

## Results

### Study participants

Participants were 25 ± 1 years old, had a BMI of 23.5 ± 0.6 kg/m^2^, and physically trained according to their VO_2peak_ (49.3 ± 2.4 ml/kg/min). Participants completed all conditions, adherence to the prescribed diet was high (97–106% of the prescribed energy intake), and attended all supervised exercise sessions (Table 1). Protein intake was significantly lower in ED-EX when compared to ED + EX,CON-EX and CON + EX (all *p* < 0.01).

**Table 1:** Energy and protein intake during each of the 4 study conditions

Condition	Energy intake (kcal/kg FFM/day)	Exercise energy expenditure (kcal/kg FFM/day)	Energy availability (kcal/kg FFM/day)	Protein intake (g/kg/day)
ED-EX	15.9 ± 0.4	0	15.9 ± 0.4	0.8 ± 0.1
ED + EX	29.7 ± 1.27	15	14.9 ± 1.0	1.4 ± 0.2
CON-EX	39.9 ± 0.6	0	39.9 ± 0.6	1.5 ± 0.1
CON + EX	52.0 ± 2.4	15	37.1 ± 2.3	1.8 ± 0.1

*ED-EX* energy deficit without exercise, *ED* + *EX* energy deficit with exercise, *CON-EX* control without exercise, *CON* + *EX* control with exercise

### Changes in body weight and composition

Significant reductions in body weight occurred in both ED conditions (Table 2). In ED-EX, losses were primarily attributed to reductions in FFM (67%), whereas in ED + EX, fat mass and FFM losses contributed almost equally.

**Table 2:** Changes in body weight and body composition over the course of each of the 4 study conditions lasting 4 days

	Body weight (kg)			Fat Mass (kg)			Fat-Free Mass (kg)		
Condition	Pre	Post	Change	Pre	Post	Change	Pre	Post	Change
ED-EX	79.6 ± 3.3	77.2 ± 3.1^***^	– 2.4 ± 0.3^aa^	7.9 ± 1.4	7.2 ± 1.2	– 0.7 ± 0.6	71.7 ± 2.3	70.1 ± 2.1	– 1.6 ± 0.8^aa^
ED + EX	80.4 ± 3.0	78.6 ± 3.0^**^	– 1.8 ± 0.4^a^	8.0 ± 1.3	7.2 ± 1.3	– 0.8 ± 0.5^a^	72.5 ± 2.5	71.5 ± 2.3	– 1.0 ± 0.8
CON-EX	79.5 ± 3.1	79.1 ± 3.1	– 0.5 ± 0.3	7.8 ± 1.4	8.2 ± 1.6	0.4 ± 0.3	71.9 ± 2.0	71.0 ± 2.0	– 0.9 ± 0.5
CON + EX	79.2 ± 3.3	79.2 ± 3.2	– 0.1 ± 0.5	7.6 ± 1.1	7.9 ± 1.3	0.3 ± 0.2	71.7 ± 2.4	71.3 ± 2.3	– 0.4 ± 0.6

*ED-EX* energy deficit without exercise, *ED* + *EX* energy deficit with exercise, *CON-EX* control without exercise, *CON* + *EX* control with exercise; *,**,*** significantly different from pre (*p* < 0.05, *p* < 0.01, *p* < 0.001; ^a, aa^: significantly different from CON (*p* < 0.05, *p* < 0.01)

### Stable nitrogen isotope ratio analysis

**Fig. 2:**
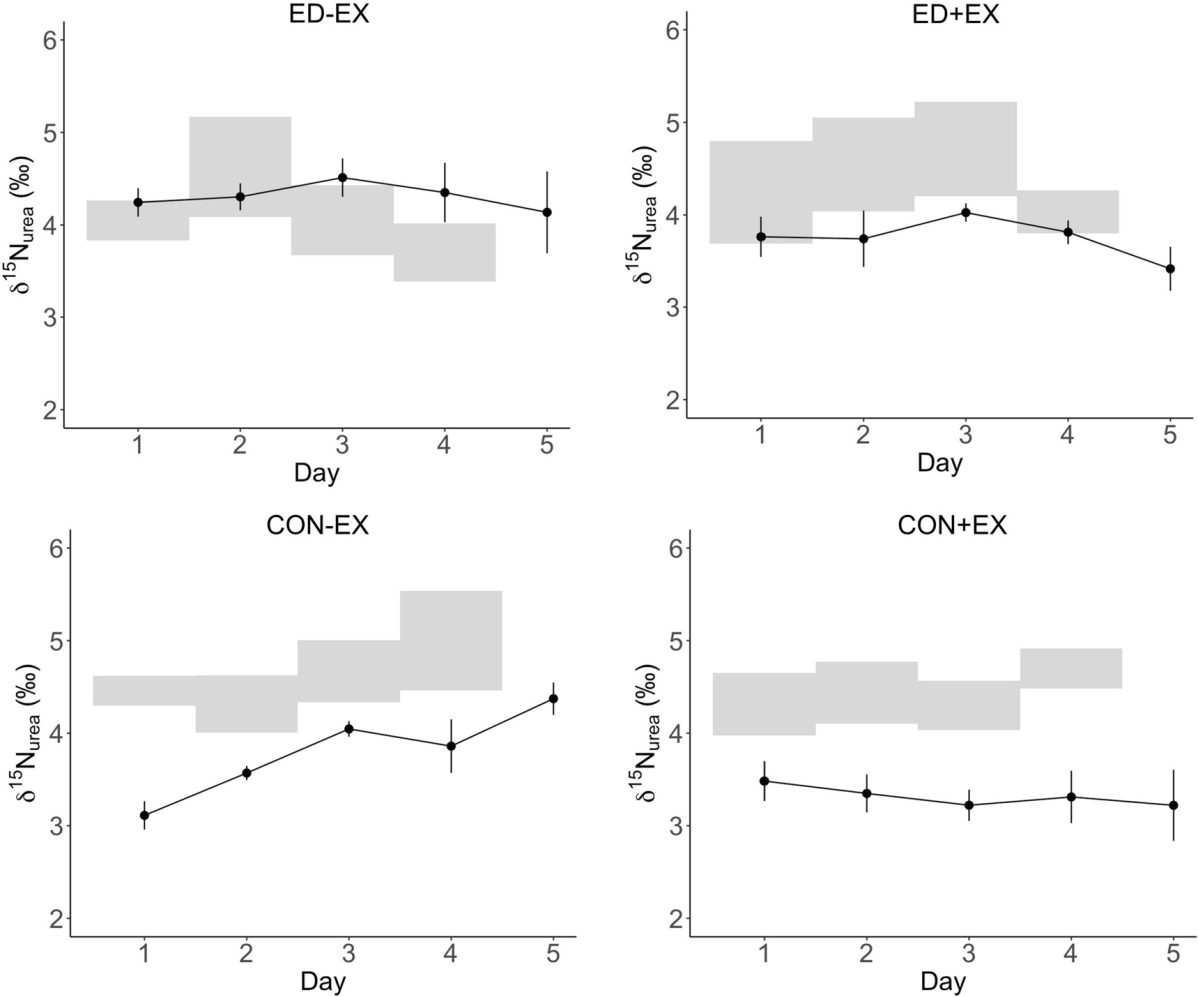
Changes in the ^15^N/^14^N isotope ratios (mean ± SEM) in urinary urea (δ^15^N_urea_) over the course of each intervention. Gray shaded areas denote the range of ^15^N/^14^N isotope ratios in the diet (δ^15^N_diet_). *ED-EX* energy deficit without exercise; *ED + EX* energy deficit with exercise; *CON-EX* control without exercise; CON + EX control with exercise

Because one participant failed to provide urine samples on days 1–3 of the ED + EX condition, δ^15^N_urea_ and Δ^15^N data are from *n* = 5 for this condition. For all other conditions, data is available for all 6 participants. As shown in Fig. 2, δ^15^N_urea_ generally followed δ^15^N_diet_. δ^15^N_urea_ increased over the course of CON-EX (*p* < 0.001) but not in any other condition. There was no significant time trend observed for Δ^15^N. We therefore aggregated isotope data across all intervention days for further analyses.

**Fig. 3:**
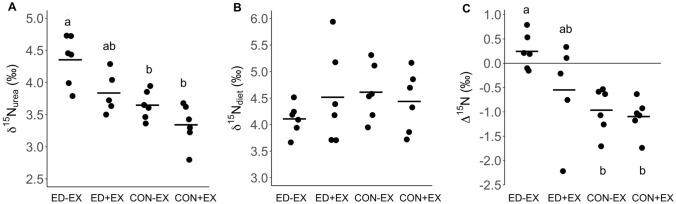
Individual ^15^N/^14^N isotope ratios in urinary urea (δ^15^N_urea_; **A**) and in the diet (δ^15^N_diet_; **B**) and the difference between urea and dietary δ^15^N (Δ^15^N; **C**) during 4 days of energy deficit without (ED-EX) and with exercise (ED + EX) and energy balance without (CON-EX) and with exercise (CON + EX). Horizontal lines represent respective group means. Labeled bars without a common letter differ, (*p* < 0.05)

Mean δ^15^N_urea_ was significantly higher in ED-EX (4.4 ± 0.2‰) when compared to CON-EX (3.7 ± 0.1‰; *p* = 0.026, *d* = 1.63) and CON + EX (3.3 ± 0.1‰, *p* = 0.001, *d* = 3.52). Mean δ^15^N_urea_ was also higher in ED + EX when compared to CON + EX (3.8 ± 0.1‰ vs. 3.3 ± 0.1‰; *p* = 0.050, *d* = 1.51). The difference in mean δ^15^N_urea_ between ED-EX and ED + EX (4.4 ± 0.2‰ vs. 3.8 ± 0.1‰; *p* = 0.050, *d* = 1.56) also trended toward significance (Fig. 3A). There were no significant differences in δ^15^N_diet_ between conditions (Fig. 3B). Consequently, Δ^15^N was positive in ED-EX (0.2 ± 0.2‰) but remained negative in ED + EX (− 0.6 ± 0.5‰) as well as both control conditions (CON-EX: − 1.0 ± 0.2‰; CON + EX: − 1.1 ± 0.2‰). When compared between conditions, Δ^15^N was significantly greater in ED-EX than in CON-EX (*p* = 0.005, *d* = 2.55) and CON + EX (*p* = 0.006, *d* = 2.31). Differences in Δ^15^N between ED + EX and CON + EX (*p* = 0.244) and between ED-EX and ED + EX (*p* = 0.244) were not significant (Fig. 3C).

### Multiple linear regression analysis

Multiple linear regression analyses showed that ED as the primary intervention explained 39% of variation in Δ^15^N (*p* = 0.026). Exercise as the secondary intervention did not meaningfully contribute to Δ^15^N and was subsequently excluded from further analysis. Dietary protein intake improved the prediction of Δ^15^N to 64% (*p* = 0.001), and the interaction between ED and dietary protein intake further improved the model (R_adjusted_ = 0.72, *p* < 0.001, Table 3). Altogether, the regression model suggests that increasing energy intake by 1 unit (1 kcal/kg FFM) reduces Δ^15^N by on average 0.076‰ and that each increase in dietary protein intake by 1 unit (1 g/day) translates into a reduction in Δ^15^N by on average 0.026‰. Consequentially, decreasing energy intake or protein intake both lead to an increase in Δ^15^N.

**Table 3:** Results from multiple linear regression analysis with Δ^15^N as dependent and protein intake (g/day), energy availability (kcal/kg FFM), and an interaction term as independent variables

Coefficient	Estimate	Error	β	t-value	p
Intercept	2.30	0.73	– 0.48	3.17	0.007
Energy availability	– 0.076	0.03	– 0.25	– 2.53	0.024
Protein intake	– 0.026	0.007	– 0.55	– 3.76	0.002
Energy availability × Protein intake	5.5 × 10^–4^	2.5 × 10^–4^	0.33	2.219	0.044

## Discussion

The present study is the first prospective analysis of δ^15^N_urea_ and Δ^15^N (calculated as the difference between δ^15^N_urea_ and δ^15^N_diet_*)* as markers of a catabolic state during controlled short-term ED in humans. As hypothesized, both δ^15^N_urea_ and Δ^15^N increased in response to ED, which is in line with previous findings of increased δ^15^N_urea_ and Δ^15^N in urine in various tissues during periods of inadequate energy intake in both animal and human studies [10–12, 14, 18, 19]. Previous studies conducted in humans are based on retrospective analysis of δ^15^N measured in hair [14, 18]. Although sampling of hair represents a non-invasive and easy sampling method, hair has a resolution of only days to weeks. In contrast, measurement of δ^15^N in urinary urea as the main end-product of amino acid catabolism allows greater resolution [21].

It is well established, that δ^15^N values of urine are generally lower when compared to other body tissues [31], which is explained by preferantion of ^14^N during trans- and deamination [9, 32]. In line, when compared to δ^15^N measured in hair, δ^15^N_urea_ appears to be lower and closer to the diet [21]. In a previous validation study, we reported δ^15^N_urea_ values within the range of 3.5–5.4 ‰ with a mean of 4.8 ‰, which were closely associated with δ^15^N_diet_ (4.1–5.4 ‰, mean: 4.6 ‰). In the present study, we determined a comparable range in δ^15^N_diet_ (3.7–5.9 ‰), with similar means within each condition. Although in the same overall range, δ^15^N_urea_ mean values were higher than δ^15^N_diet_ in ED-EX (4.4 ± 0.2‰ vs. 4.1 ± 0.2‰) and on average only about 0.7‰ below δ^15^N_diet_ in ED + EX (3.8 ± 0.1‰ vs. 4.5 ± 0.4‰). In contrast, the gap between δ^15^N_urea_ and δ^15^N_diet_ was almost 1‰ in CON-EX (3.7 ± 0.1‰ vs. 4.6 ± 0.2‰) and CON + EX (3.3 ± 0.1‰ vs. 4.4 ± 0.2‰). Thereby, we demonstrate that a short-term energy deficit is associated with increased δ^15^N_urea_ values, which are closer to or even exceed δ^15^N_diet_, resulting in an increased (“less negative” or even positive) Δ^15^N. These findings are also in line with previous results from animal studies reporting increases in δ^15^N in urine or urinary urea during periods of inadequate energy (and protein) intake [11, 19, 33, 34]. It has been suggested that changes in δ^15^N could be indicative of negative nitrogen balance and increased loss of body protein [5]. Indeed, results from animal studies confirm this by showing a positive relationship between losses of lean mass and increasing δ^15^N values in urine [34]. In humans, studies analyzing δ^15^N in hair of anorexia nervosa patients showed elevated values which decreased again during rehabilitation [13]. Similarly, weight loss due to morning sickness in pregnant women was also traceable as increased δ^15^N in hair samples [18]. Although our experiment was not suited to establish a direct relationship between loss of body protein and δ^15^N_urea_, we observed a negative trend between δ^15^N_urea_ and changes in body weight (*r* = − 0.62, data not shown). In addition, due to available data on δ^15^N_diet_, we were also able to calculate Δ^15^N, which was not possible in previously mentioned human studies.

Overall, it appears that isotopic fractionation is complex and δ^15^N as well as Δ^15^N not only vary between tissues [35], but also change in response to an energy deficit. In fact, in a tightly controlled animal study employing controlled calorie restriction in rats, Huneau et al. demonstrated in vivo, that changes in isotopic signatures can be viewed as “*fingerprints*” of AA flux directed toward oxidation or protein synthesis. In their study, the authors report increases in Δ^15^N in urine and tissues such as the liver, with a concomitant decrease in Δ^15^N in skeletal muscle. Based on a previously published model [20], the authors suggested that these contrasting findings are due to maintained, preferable elimination of ^14^N AAs in tissues such as the liver, while the muscle, which represents the largest reservoir of AAs, releases proportionally more ^15^N-enriched AAs, resulting in a decrease in Δ^15^N. Notably, in the above mentioned study, the increase in Δ^15^N in urine was associated with a loss of liver protein mass [19]. Although we cannot confirm the role of protein losses from tissues to increases in δ^15^N_urea_ and Δ^15^N, respectively, we explain the increase by means of increased oxidation of AAs and preferable elimination of ^14^N in the liver, which supports increased demand for substrates for gluconeogenesis during an energy and carbohydrate deficit [20]. The preferable elimination of ^14^N AAs would also fall in line with the “anabolic model” described by Lee et al. This model postulates that during periods of catabolism, the excretion of ^14^N results in the release of proportionally more ^15^N AAs into the free AA pool, which are subsequently used for tissue protein synthesis [34]. Yet, the current study does not allow us to draw conclusions about δ^15^N of tissues. Considering that our study period was only 4 days, we acknowledge that participants were neither in an isotopic nor an elemental steady state condition. For the analysis of δ^15^N_urea_ and Δ^15^N as prospective markers with timely resolution, a steady state was not desirable.

However, we can be certain that the participants were all in negative energy balance, which we could verify indirectly through the noticeable weight loss in both ED conditions. While weight loss was similar in the two ED conditions, the composition of weight loss was impacted by exercise. While 67% of weight loss during the ED-EX condition was attributed to loss of FFM, only ~ 50% were attributed to FFM in ED + EX. The difference of approximately 17% falls in line with estimations from Weinheimer et al. [3]. Given the protective effect of exercise on FFM, it is speculated that exercise could attenuate the decrease in Δ^15^N in skeletal muscle, as a consequence of reduced loss of skeletal muscle mass.

In addition to exercise, protein intake is an important modulator of AA metabolism and high-protein diets are commonly prescribed to maximize retention of FFM during weight loss. While energy intake was tightly controlled, we did not control for protein quantity, resulting in comparable intakes between the CON and ED + EX condition, but a lower intake in ED-EX. Although we acknowledge the lack of standardized protein intake as limitation, we accounted for quality of the diet and estimated δ^15^N_diet_ which allowed calculation of Δ^15^N. The importance of taking δ^15^N_diet_ into account is necessary, as we already reported differing δ^15^N_urea_ values in response to different protein sources such as meat or seafood [21].

Finally, we acknowledge that our analysis had a small sample size and was exploratory in nature. Hence, further tightly controlled studies are needed to evaluate the magnitudes of changes, which could be used as clinically relevant changes in δ^15^N_urea_ and Δ^15^N, respectively. In a clinical setting, such biomarkers could be useful in the monitoring of patients at risk for cachexia or other scenarios of rapid weight loss. As previously conducted with δ^15^N from hair, δ^15^N_urea_ and Δ^15^N could also be used to monitor patients with anorexia.

## Conclusion

Our findings show that δ^15^N_urea_ and Δ^15^N increase during a period of catabolism and are indicative of the metabolic state. We are the first to report a measurable increase in δ^15^N_urea_ and Δ^15^N after as little as 4 days of ED. We propose that the increase is the result of increased AA oxidation and preferable elimination of ^14^N, likely in the liver. The potential of δ^15^N and Δ^15^N as prospective markers of catabolism need to be further evaluated in studies with fixed protein intake. In addition, the impact of exercise needs further clarification.

## Data Availability

The raw data of this article will be made available by the authors, upon reasonable request.
